# Supplementary material for: Stretchable Transparent Light-Emitting Diodes Based on InGaN/GaN Quantum Well Microwires and Carbon Nanotube Films
Source: Nanomaterials (Basel). 2021 Jun 7;11(6):1503. doi: 10.3390/nano11061503 (PMC8230151; doi:10.3390/nano11061503)
Supplement: Supplementary file 1 [file nanomaterials-11-01503-s001.zip › nanomaterials-1213661-supplementary.pdf]

Supplementary Materials

Stretchable Transparent Light-Emitting Diodes Based on InGaN/GaN Quantum Well Microwires and Carbon Nanotube Films

Verification of Strain-Insensitive SWCNT Contact Properties

Electrical properties of SWCNT contacts were verified for two types of SWCNT application onto PDMS 2 cm long films. The first type was SWCNT contact stripes applied to pre-stretched by 10% (~2 mm), the second type was applied to relaxed (i.e. not pre-stretched) PDMS films. Schematic image shown in Figure S1. The representative results of conductivity verification are shown in Figure S2.

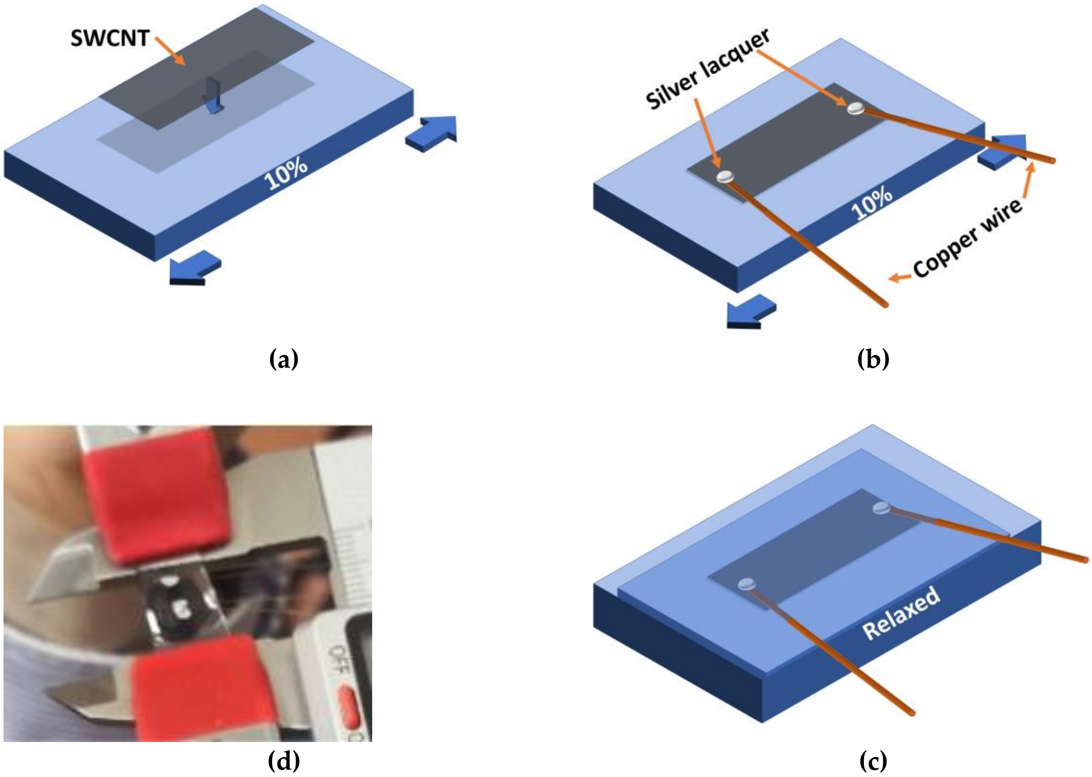

Figure S1. Schematic image of electrical contacting to a PDMS film: (**a**) application of SWCNT contact pad onto 10% pre-stretched PDMS film, (**b**) connection to the SWCNT contact pad with copper wires and silver lacquer, (**c**) relaxed PDMS film with applied contacts buried into PDMS, (**d**) image of PDMS film with electrical contacts.

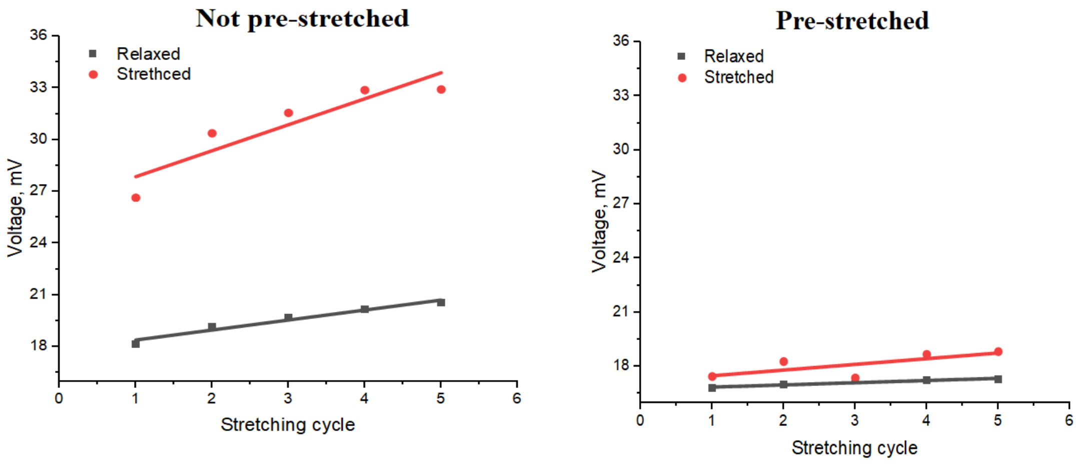

(a) **(b)**

Figure S2. Representative voltage at constant current for (**a**) a not pre-stretched SWCNT sample and (**b**) a pre-stretched sample, measured in stretching conditions (red lines) and relaxed state (black lines). Both sample types had similar conductivity in relaxed state, however, in stretching conditions the pre-stretched samples had significantly lower resistivity and degradation due to the stretching.

As it is shown in Figure S1, the voltages of the pre-stretched sample and the not pre-stretched sample are similar in relaxed state, namely it is about 17 and 19 mV, respectively. However, the voltage of the pre-stretched sample changed significantly less than for the not pre-stretched sample, measured in stretching conditions: it is about 18 mV and 30 mV, respectively. Moreover, the pre-stretched sample showed almost no degradation because of stretching (less than 5%), while the not pre-stretched sample had a 20% degradation after 5 stretching cycles.

We conclude that the SWCNT films applied to a pre-stretched PDMS sample retained conductivity in stretching conditions, so the strain-insensitive LED could have increase of the working voltage after stress test mainly due to the SWCNT/MW interface, and not due to the SWCNT film deterioration.
